# Nationwide Description of Live Japanese Births by Day of the Week, Hour, and Location

**DOI:** 10.2188/jea.12.330

**Published:** 2007-11-30

**Authors:** Noriko Morita, Noriko Matsushima, Nozomi Ogata, Keigo Saeki, Mariko Ishibashi, Hideki Komukai, Ryozo Matsuda, Norio Kurumatani

**Affiliations:** Department of Hygiene, Nara Medical University.

**Keywords:** live births, obstetric intervention, spontaneous delivery, temporal variations

## Abstract

To characterize temporal variations of live births in Japan, we analyzed data on the 1,203,147 births of 1998. In hospitals, with 20+ beds, the daily average of live births was significantly lower at weekends and national holidays (mean=1,433, SD=100) than on weekdays (mean=1,957, SD=126). Hourly distributions of live births showed a single sharp peak at 1:00-2:59 pm on weekdays with a small peak at an earlier hour on Saturdays, Sundays and national holidays. The results in clinics, with no bed or less than 20 beds, were similar to those in hospitals except on Saturdays. The difference in the daily average of live births between Saturdays and weekdays was smaller in clinics than that found in hospitals, and hourly distributions on Saturdays resembled those of weekdays but not Sundays or national holidays. Maternity homes showed no differences in the mean number of daily live births over the days of the week including national holidays, and no clear peak of percentage distributions of hourly live births on each day of the week. The present study suggests that the weekly and hourly variations observed in hospitals and clinics are not due to a biological rhythm of labor, but to obstetric intervention in the timing of delivery, either through induction of labor or elective cesarean section.

In the early 1950s in Japan, almost all deliveries occurred at home, but in the 1960s deliveries in hospitals and those in clinics, legally defined as a medical facility having no bed or less than 20 beds, increased steeply.^1^ The number of live births in 1975 delivered in hospitals and clinics accounted for 90% of all live births, and in recent years the figure is almost 99%,^2^ coupled with marked advances in labor management. The advent of oxytocin and prostaglandin, which makes the timing of deliveries more predictable,^3^^,^^4^ can affect the distribution of live births by day of the week or time of day. Studies in Japanese hospitals^5^^-^^9^ found the significantly lower number of live births on Saturdays and Sundays than on the other days of the week. Such results suggest that spontaneous deliveries might be intervened by obstetric practices. However, there have only been a few studies and those results were based on data in limited medical institutions such as university hospitals^5^^,^^6^ or in cities.^7^^,^^8^ Therefore, those findings may not represent the nationwide pattern. In addition, the characteristics of live birth distributions in hospitals and clinics can be shown much more clearly when compared with those in non-medical delivery facilities. In Japan we have maternity homes where midwives alone provide sole assistance in childbirth and observe the pregnancy/puerperal course. However, to date no reports have been published on live birth distribution in the homes. Although only 1% of all live births are delivered at maternity homes in recent years,^2^ more than 10,000 live births a year are large enough to be examined statistically.

The present study aimed at investigating the distribution of live births by day of the week, hour and location, using a nationwide single annual sample in Japan, then discussing the effects of obstetric intervention on deliveries.

## MATERIALS AND METHODS

At the Statistics and Information Department of the former Ministry of Health and Welfare in July 2000, we obtained papers printed with data on the number of live births nationwide since 1981. These data, not published but kept in the Department, can be photocopied with permission.^10^ The latest data, all the births in 1998, was used for the present study. The numbers of live births were tabulated by location (hospitals, clinics, maternity homes, own homes and others), date and time of birth applying one-hour intervals. All figures were inputted into a computer and used in the following analysis.

The 365 days in 1998 were classified into 8 categories: national holidays and each day of the week from Monday to Sunday. When the day, including Sunday, was a national holiday, it was classified as a national holiday. Substitute holidays were also considered to be national holidays. As is custom in Japan, most enterprises and medical institutions are closed at the end and beginning of the year; therefore, 3 days of the new year and 3 days on the end of the year were included in the national holiday category in the present study. As a result, there were 20 national holidays, 49 Mondays, 48 Tuesdays, 47 Wednesdays, 50 Thursdays, 51 Fridays, 49 Saturdays, and 51 Sundays. The t-test was applied to examine the difference in the means of daily live births between weekdays in total and the remaining days in total. The Tukey’s HSD test^11^ was utilized in an attempt to reveal a difference in the daily average of live births in each combination of weekdays and between Saturdays, Sundays and national holidays. The t-test was also used for the comparison between the average of daily live births on the 365 days of the year and that of national holidays or each day of the week. P values of less than 0.05 were considered statistically significant.

## RESULTS

### Places of birth

The number of live births in Japan in 1998 was 1,203,147. Those born in hospital accounted for 651,323 (54.1%), and 537,752 (44.7%) were born in clinics, together accounting for 98.8% of all live births. Only 11,932 (0.99%) were born in maternity homes. Live births in own homes and other places accounted for 1,868 (0.16%) and 272 (0.02%), respectively. They were excluded from further analysis because of the small numbers.

### Hospitals

**Figure 1.**
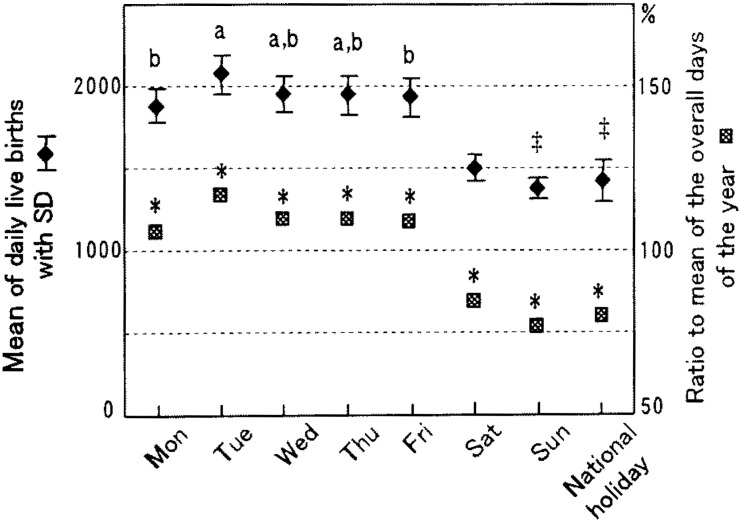
Mean number of daily live births by day of the week and ratios of live births on each day to the overall daily average in hospitals in 1998, Japan. a and b show significant differences at p<0.05 in the mean of the day compared with those of Mondays and Tuesdays, respectively, using the Tukey’s HSD test among weekdays only. ‡ shows a significant difference at p<0.05 in the mean of the day compared with that of Saturdays using the Tukey’s HSD test among Saturdays, Sundays and national holidays. * shows a significant difference in the mean of the respective day compared with the overall daily average for the year.

The overall daily average of live births in 1998 was 1,784 (SD=274). However, differences were observed among the days of the week. Tuesdays were the highest at 2,072 (SD=113), and Sundays were the lowest at 1,372 (SD=62) (Figure 1). The daily average of weekdays in total was 1,957 (SD=126). When the daily average was compared in each combination of weekdays, significant differences were observed in all except those combinations of Fridays and Mondays, Wednesdays or Thursdays, and between Wednesdays and Thursdays. The daily average of live births on Saturdays, Sundays and national holidays was 1,433 (SD=100), which was significantly lower than that of weekdays (1,957). Comparing Saturdays, Sundays and national holidays showed a significantly large number of live births on Saturdays than on Sundays or national holidays, but no difference between Sundays and national holidays. When the mean number of live births on each day of the week was compared with the overall daily average of the year, as illustrated by the marks corresponding to the right axis in Figure 1, the number on each weekday was significantly higher (8-16%) than that for all days of the year. On Saturdays, Sundays, and national holidays it was significantly lower (16-23%).

**Figure 2.**
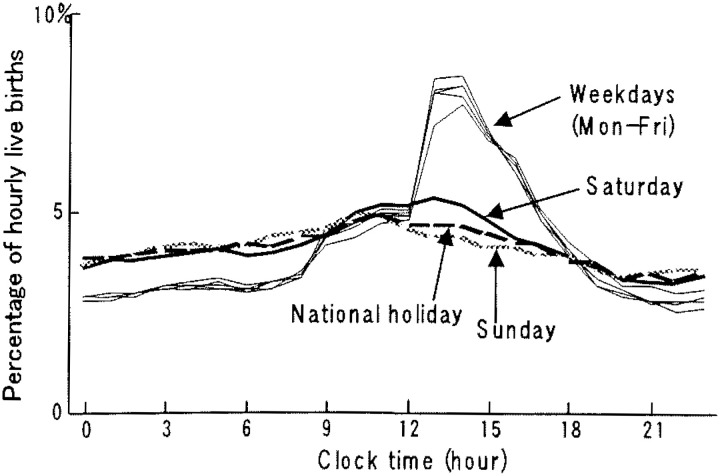
Hourly percentage of daily live births by day of the week in hospitals in 1998, Japan.

Figure 2 shows the percentage distribution of the hourly live births. On weekdays the percentage increased at 9:00-9:59 am, showing a single sharp peak at 1:00-2:59 pm, and gradually decreased thereafter. It was lowest at 10:00-10:59 pm. On Sundays and national holidays, the percentage gradually reached a small peak at 11:00-11:59 am, gradually decreased, and was lowest at 9:00-9:59 pm. On Saturdays, a small peak was observed at 1:00-1:59 pm, but as a whole, the percentage distribution was similar to those of Sundays and national holidays.

### Clinics

**Figure 3.**
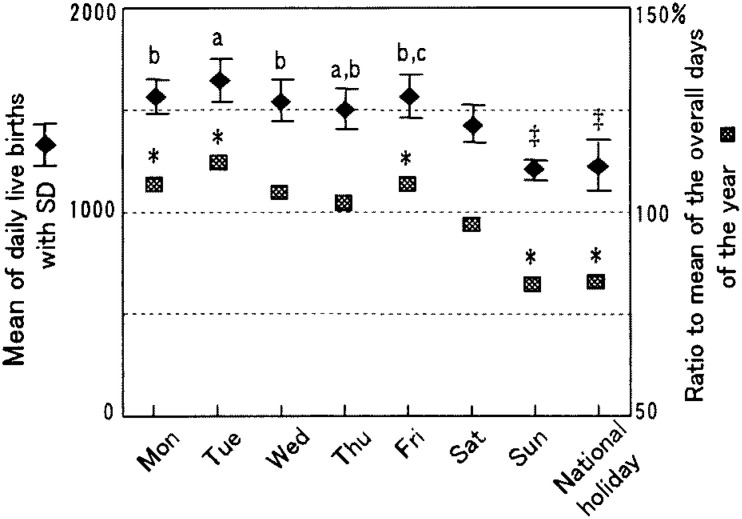
Mean number of daily live births by day of the week and ratios of live births on each day to the overall daily average in clinics in 1998, Japan. a, b and c show a significant differences at p<0.05 in the mean of the day compared with those of Mondays, Tuesdays and Thursdays, respectively, using the Tukey’s HSD test among weekdays only. ‡ shows a significant difference at p<0.05 in the mean of the day compared with that of Saturdays using the Tukey’s HSD test among Saturdays, Sundays and national holidays. * shows a significant difference in the mean of the respective day compared with the overall daily average for the year.

The overall daily average of live births in 1998 in clinics was 1,473 (SD=170). Similar to hospitals, the daily average was highest (1,640 with SD=102) on Tuesdays and lowest (1,201 with SD=44) on Sundays (Figure 3). The daily average of weekdays in total was 1,561 (SD=107). No difference was observed between Mondays and Wednesdays, or Fridays, or between Wednesdays and Thursdays, but significant differences were observed between the days of the week in all the other combinations. As in hospitals, the daily average for Saturdays, Sundays and national holidays (1,295 with SD=131) was significantly lower than that of weekdays (1,561). Comparing Saturdays, Sundays and national holidays showed there was a significantly higher mean number of live births on Saturdays than on Sundays or national holidays. Similar results were also found in hospitals, but a smaller difference was observed in the mean number between Saturdays (1,424) and weekdays (1,561) in clinics than in hospitals (1499 vs 1957). No significant difference was obtained between Sundays and national holidays. When compared with the daily average of live births over all the days of the year, as in Figure 3, those on Mondays, Tuesdays and Fridays showed 6-11% significantly higher values and those on Sundays and national holidays 17-19% significantly lower values. However, no difference was observed for the remaining three days.

**Figure 4.**
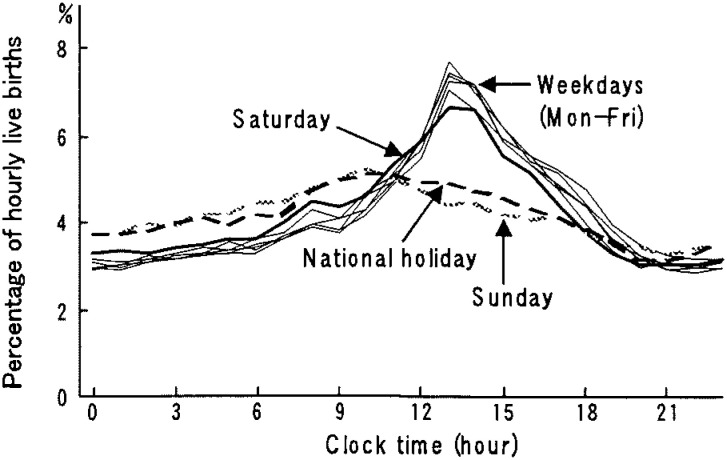
Hourly percentage of daily live births by day of the week in clinics in 1998, Japan.

Figure 4 shows the percentage distribution of the hourly live births on each day of the week. On weekdays, the percentage began to gradually increase from 7:00-7:59 am, reaching a single sharp peak at 1:00-1:59 pm as in hospitals, and subsequently decreasing to its lowest at 9:00-9:59 pm. On Sundays and national holidays, the percentage reached a peak at 10:00-10:59 am and was lowest at 9:00-9:59 pm. The distribution on Saturdays showed a pattern similar to those of weekdays but not to those of Sundays and national holidays.

### Maternity homes

**Figure 5.**
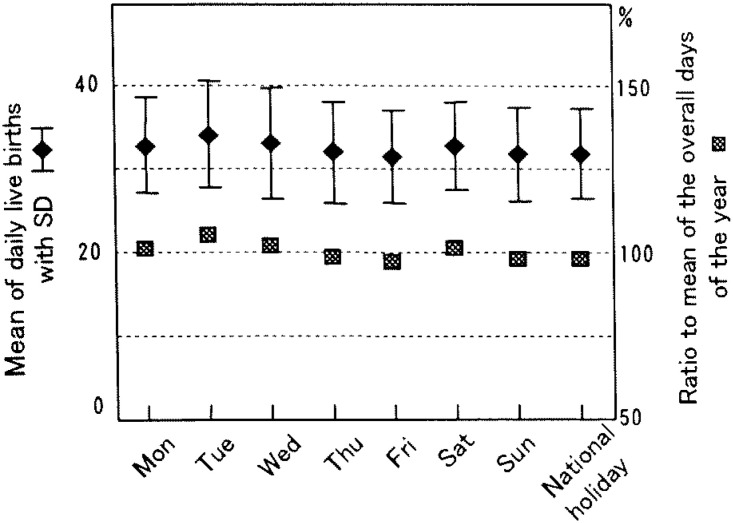
Mean number of daily live births by day of the week and ratios of live births on each day to the overall daily average in maternity homes in 1998, Japan.

The daily average of live births for the overall days of the year in maternity homes was 33 (SD=6). The mean number was the highest (34 with SD=6) on Tuesdays and was the lowest (32 with SD=6) on Fridays (Figure 5), but unlike hospitals and clinics no statistical differences were obtained in any combination among weekdays. In addition, the average of live births for Saturdays, Sundays and national holidays (32 with SD=5) did not significantly differ from that of weekdays (33). No differences were observed between the daily average of live births on the overall days of the year or that of any day of the week, either.

**Figure 6.**
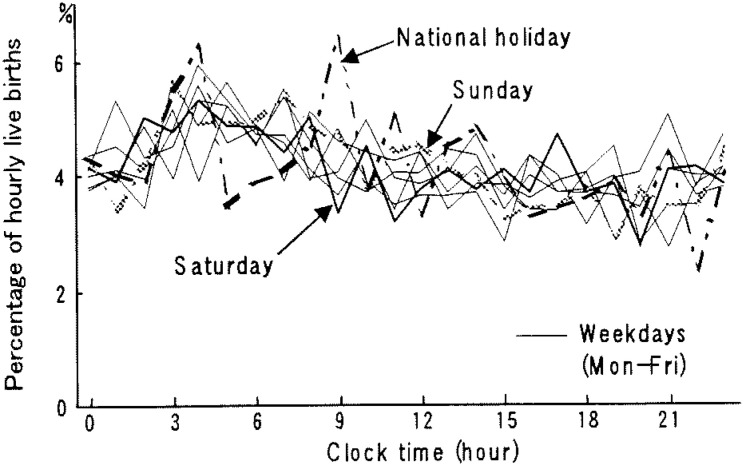
Hourly percentage of daily live births by day of the week in maternity homes in 1998, Japan.

As shown in Figure 6, the percentage distribution of hourly live births showed fluctuations because of the small number of live births per hour per day. In contrast to those in hospitals and clinics, however, temporal variations on each day of the week including national holidays showed a similar pattern: higher in the early morning and lower in the evening.

## DISCUSSION

We could not analyze data on the number of stillbirths in the same manner as we have done in the current study because the data was not provided by the former Ministry of Health and Welfare. A total of 38,980 stillbirths were reported in 1998.^12^ Of all, 17,450 occurred in hospitals, 21,222 in clinics and 35 in maternity homes. These figures corresponded to around 3% or less of live births in each facility. The percentages were small and unlikely to change the present results substantially.

Midwives in Japan are licensed by the national government. They are, however, prohibited from making diagnoses using apparatus, performing episiotomies, and administering drugs including oxytocin and prostaglandin.^13^ To induce and promote labor, papillary massage, thermotherapy and guidance in walking are performed,^14^ but they are less effective and less accurate than drugs. When medical treatment is required due to premature rupture of membranes, decreased fetal heart rates or other conditions, women are transported to medical institutions. Accordingly, delivery in maternity homes is considered to be the place where labor is spontaneous with no obstetric intervention. Under such a situation deliveries occurred evenly over all of the days of the week (Figure 5), with deliveries being frequent in the early morning rather in the afternoon (Figure 6). These findings are consistent with a result obtained for about 21,000 deliveries from spontaneous labor in 18 hospitals in Tokyo.^5^

Only a few studies^5^^-^^9^ have been done for temporal variation of live births in hospitals in Japan. All of them showed similar results, although those were based on much smaller cases in a limited area. For example, Esaki et al.^7^ evaluated about 3,500 cases over three years since 1981 in a middle sized city located in west Japan. Ando^5^ analyzed about 3,200 cases collected for five years since 1988 from 18 medical institutions, mainly university hospitals in Tokyo. Esaki et al.^7^ observed that the daily number of live births on weekdays was three times larger than that of national holidays. Ando,^5^ not considering national holidays, indicated that live births occurred frequently on Tuesdays and Fridays and less on Saturdays and Sundays. The study also indicated that the percentage distribution of hourly live births showed a single sharp peak between 2:00 and 3:59 pm. The present study, using a nationwide whole sample of about 650,000 live births in hospitals, supported these earlier findings and precisely indicated the temporal variations of live births in hospitals. Namely, unlike in maternity homes, the number of live births in hospitals varied among days of the week: higher on weekdays and much lower on Saturdays, Sundays and national holidays (Figure 1), and showed a single sharp peak at 1:00-2:59 pm on weekdays (Figure 2).

The results obtained in clinics were the same as in hospitals except on Saturdays. The difference in the daily average of live births between Saturdays and weekdays was smaller than that of hospitals (Figure 3), and the hourly variation of live births on Saturdays resembled those of weekdays but not Sundays or national holidays (Figure 4). No reports have pointed out these findings. One of the reasons might be that a majority of clinics, unlike hospitals, are private and open on Saturdays in Japan, although further study is necessary on this point.

A study conducted on 900,000 live births throughout Australia^15^ reported that the mean number on weekdays, particularly on Thursdays, was about 10% higher, but on Sundays and holidays it was about 25% lower than the overall daily average. In a study of 65,000 births in Wisconsin, U.S.A.,^16^ the mean number of live births on Tuesdays was about 9% higher while on Sundays and holidays it was 15-19% lower than the overall daily average of the year. In England and Wales,^17^ live births were reported to be 7% higher on weekdays but 8-23% lower on Sundays and national holidays than the overall daily average. Although these studies^15^^-^^18^ did not show the results relating to location of birth, all of the authors stated that the weekly patterns of live births was closely associated with an increasing prevalence of obstetric intervention. The results shown in these countries were in accordance with our results in hospitals and clinics. In addition to this, the fact that the temporal patterns of live births were completely different from those in maternity homes suggests that deliveries in hospitals and clinics occurred not because of a biological rhythm but rather obstetric practices such as induction of labor or elective cesarean section. Further studies are necessary, however, to confirm the association between the practices and live birth distributions.

In 1993, an association of obstetricians in Japan^19^ announced medical indications for labor induction and recommended that delivery be performed during the day on weekdays. Besides, it is realistic for obstetricians to avoid delivery during the night and on weekends or holidays when staff are fewer and cannot easily assemble, even in an emergency or sudden changes in the condition of pregnant women.^3^ The operation days and times are usually fixed in medical institutions, especially in hospitals.^6^ Cesarean sections are planned on these occasions and these in turn contribute to a cluster of live births on certain weekdays and certain times.^3^^,^^5^^,^^20^ According to the Medical Facility Survey conducted nationwidely in a three-year interval, the percentage of cesarean sections performed for live births has been increasing in recent years in Japan.^21^ Those are 17.4% and 11.4%, respectively, in hospitals^22^ and clinics^23^ in 1999, the next year whose data we dealt with. These practices observed for cesarean sections may contribute to the weekly pattern of the large number on weekdays and fewer on Sundays and national holidays, and the hourly pattern of a single sharp peak in the early afternoon both in hospitals and clinics (Figures 1 to 4). In both facilities Tuesdays showed a significantly higher mean number of live births than that of other weekdays. These results imply that procedures accelerating spontaneous onset of labor such as metreurysis, amniotomy and prescribing prostaglandin E2 might commonly be conducted on Mondays rather than on Sundays.

Thus, the present nationwide analyses clarified that the weekly and hourly patterns of live births in both hospitals and clinics were different from those of maternity homes, where deliveries are spontaneously labored. It also suggested that the patterns might be due to obstetric interventions. This association can be clear by time trend analysis of patterns of live birth distributions with changes in utilization of obstetric interventions. A further study is expected on this point.
